# DYRK2 Negatively Regulates Cardiomyocyte Growth by Mediating Repressor Function of GSK-3β on eIF2Bε

**DOI:** 10.1371/journal.pone.0070848

**Published:** 2013-09-04

**Authors:** Celine S. Weiss, Marco M. Ochs, Marco Hagenmueller, Marcus R. Streit, Pratima Malekar, Johannes H. Riffel, Sebastian J. Buss, Karl H. Weiss, Junichi Sadoshima, Hugo A. Katus, Stefan E. Hardt

**Affiliations:** 1 Internal Medicine III, University Hospital Heidelberg and DZHK (German Center for Cardiovascular Research), Heidelberg, Germany; 2 Internal Medicine IV, University Hospital Heidelberg, Heidelberg, Germany; 3 New Jersey Medical School, University of Medicine and Dentistry of New Jersey, Newark, New Jersey, United States of America; Ospedale Pediatrico Bambino Gesu', Italy

## Abstract

**Background:**

A prerequisite of hypertrophic response of the myocardium is an increase in protein synthesis. A central regulator of translation initiation is Eukaryotic initiation factor 2B (eIF2B). Here we assessed the hypothesis that regulation of protein synthesis via eIF2Bε is essential to cardiac hypertrophic response in vivo.

**Methods:**

Two transgenic mouse lines were generated with cardiac restricted overexpression of eIF2Bε or its mutant eIF2Bε-eIFS^535^A, which cannot be inactivated by phosphorylation through GSK-3β.

**Results:**

(1) Under baseline conditions eIF2Bε transgenic mice showed no difference in cardiac phenotype compared to wild type, whereas in the mutant eIF2Bε-S^535^A an increase in LV/tibia length (7.5±0.4 mg/mm vs. 6.2±0.2 mg/mm, p<0.001) and cardiomyocyte cross sectional area (13004±570 vs. 10843±347 RU, p<0.01) was observed. (2) Cardiac overexpression of eIF2Bε did not change the response of the heart to pathologic stress induced by chronic isoproterenol treatment. (3) Cardiac overexpression of the eIF2Bε transgene was followed by overexpression of DYRK2 which is known to prime the inhibitory action of GSK-3β on eIF2Bε, while DYRK1A and GSK-3β itself were not increased. (4) In C57BL/6 mice after 48 h of isoproterenol-stimulation or aortic banding, eIF2Bε was increased and DYRK2 was concomitantly decreased. (5) In line with these in vivo findings, siRNA knockdown of DYRK2 in cultured cardiomyocytes resulted in decreased levels of p(S535)- eIF2Bε, (6) whereas adenoviral induced overexpression of DYRK2 was accompanied by clearly increased phosphorylation of eIF2Bε, indicating a coordinated response pattern (7) Adenoviral induced overexpression of DYRK2 leads to significantly reduced cardiomyocyte size and diminishes hypertrophic response to adrenergic stimulation.

**Conclusions:**

The interaction of GSK-3β and its priming kinase DYRK2 regulate the activity of eIF2Bε in cardiac myocytes. DYRK2 is a novel negative regulator of cardiomyocyte growth. DYRK2 could serve as a therapeutic option to regulate myocardial growth.

## Introduction

Left ventricular hypertrophy (LVH) is an important risk factor for ischemia, arrhythmia and sudden death, independent of the underlying hypertrophic stimulus [1]. Given the limited ability of current pharmacological principles to prevent or regress LVH, novel therapeutic strategies are urgently needed to prevent LVH and its progression rather than to treat established cardiomyopathy and heart failure. The hypertrophic growth of the heart results primarily from an increase in cardiomyocyte size and not from proliferation [2].Hence, the regulation of overall rates of protein synthesis in cardiomyocytes is the primary determinant for the growth associated with LVH. Anyhow, the molecular mechanisms by which protein synthesis and cardiac growth is regulated have received little attention so far.

We have previously shown that the guanine nucleotide exchange factor 2B is one of these crucial regulators of protein synthesis and cardiomyocyte growth in vitro [3]. eIF2B is a guanine nucleotide exchange factor which mediates the exchange of GDP bound to translation initiation factor eIF2 for GTP. This exchange process is a key regulatory step for the control of translation initiation in all eukaryotic organisms and increases thereby protein synthesis. eIF2B is composed of five subunits termed α-ε in order of increasing size [4]. The 82kDa ε-subunit is the predominant regulative subunit and carries out the guanine nucleotide exchange [5]. Subunits α, β and δ of eIF2B form a regulatory subcomplex that binds to phosphorylated eIF2α and down-regulate the activity of eIF2Bε [6] while eIF2Bγ enhances the activity of the ε-subunit [7].

The activity of eIF2B is highly controlled in many different cell systems in response to a variety of cellular conditions: Stressful situations (e.g. viral infections) decrease its activity, whereas stimulation of cells or tissues by insulin, growth factors or mitogens leads to its activation [8], [9]. eIF2B is constitutively active and all known regulatory mechanism have exclusively inhibitory effects [10]. It can be controlled directly through changes in its phosphorylation status, or indirectly via phosphorylation of eIF2, since eIF2 (αP) is a potent competitive inhibitor of eIF2B (see for review [7]). Glycogen synthase kinase 3β (GSK-3β) is thought to play the greatest role in the inhibition of the activity of eIF2Bε by phosphorylating on Serine 535 [10], [11]. Previously, phosphorylation of the ε-subunit of eIF2B by GSK-3β was identified in vitro in cardiomyocytes as an important signalling step mediating the antihypertrophic actions of GSK-3β through inhibition of protein translation [3].

GSK-3β usually requires for interaction with its substrates like eIF2B a phosphorylated serine or threonine residue located four amino acids C-terminal to its phosphorylation site [11]. In this context, DYRK 1A and 2 have been described to physically interact with eIF2Bε in vitro, but their physiological role especially in cardiac hypertrophy still remains unclear. Following priming activity by DYRK2, the phosphorylation rate of tau by GSK-3β was 8 fold increased [11]. Even greater effect on the phosphorylation rate of Glycogen Synthase by GSK-3β was reported [12]. Phosphorylation of eIF2Bε on serine 535 by GSK-3β was only seen, when it has been previously phosphorylated by DYRK2 [12].

The DYRK family of protein kinases (dual specificity tyrosine(Y)-phosphorylation regulated kinase) are pleiotropic regulators of cellular functions and are evolutionary highly conserved [13]. In mammalians seven isoforms are known (DYRK1A, DYRK1B, DYRK1C, DYRK2, DYRK3, DYRK4A, DYRK4B). The sequences highly equal themselves in their kinase domain and the DYRK homology box [11]. DYRK1A is ubiquitously expressed in mammalians, whereas the expression of all other isoforms follows a tissue specific manner. Little is known yet about potential regulation mechanisms of DYRK kinases. They activate themselves during protein synthesis by autophosphorylation of a tyrosine residue in their activating domain [11]. After this step they act as constitutively active serine/threonine kinases. The autophosphorylation site is the only phosphorylation site described so far. DYRK2 is localized in the cytoplasm, whereas DYRK1A is predominantly found in the nucleus [3], [11]. According to this distribution both kinases mainly phosphorylate substrates in their specific compartment.

In our previous work we could demonstrate that eIF2Bε is a potent stimulator of hypertrophic growth in cultured cardiomyocytes by increasing protein synthesis rate. In order to evaluate eIF2Bε as a therapeutic target for the treatment of cardiac hypertrophy we further investigated the related signalling mechanisms. This study is the first to show that the interaction of GSK-3β and its priming kinase DYRK2 is a potent regulatory mechanism of the activity of eIF2B and cardiac hypertrophy in vivo.

## Materials and Methods

### Generation of transgenic mice

To investigate the physiological role of eIF2Bε for myocardial hypertrophic response in vivo, we generated two transgenic mouse lines with cardiac restricted overexpression of eIF2Bε (eIF2Bε-TG), or overexpression of a mutant eIF2Bε-S^535^A (eIF2Bε-S^535^A-TG) which cannot be inactivated by phosphorylation through GSK-3β. cDNA encoding eIF2Bε or eIF2Bε-S^535^A was cloned into a plasmid containing the heart-specific α-myosin heavy chain (MHC) promoter and human growth hormone (hGH) poly(A)^+^ signal. DNA injections were performed into C57BL/6 pronuclei. Genomic DNA was isolated from mouse tail biopsies and analyzed by PCR with primers specific for the hGH poly(A)^+^ signal: (forward: 5′- GTC TAT TCG GGA ACC AAG CTG GAG TG-3′, reverse: 5′- ACA GGC ATC TAC TGA GTG GAC CCA AC-3′). Cardiac-specific overexpression of eIF2Bε was confirmed by Western blotting. Generation of transgenic animals as well as animal handling and experiments were performed according to the institutional guidelines of the University of Heidelberg and were approved by the animal experiment review board of the government of the state of Baden-Württemberg, Germany (approval number: 35-9185.81/G-76/07). All animals analyzed were males at the age of 3–4 months unless stated otherwise. Controls were performed with male littermates.

### Echocardiography

Mice were anesthetized by isoflurane inhalation (∼2% isoflurane), positioned on a warming pad (Fine Science Tools GmbH, Heidelberg, Germany) and allowed to breathe spontaneously. Echocardiography was performed by using an HDI 5000 CV echocardiography machine (ATL Ultrasound, Philips, Bothell, WA, USA) equipped with a 10-MHz probe. M-mode measurements of left ventricular dimensions were averaged from at least three cycles, using the edge–to–leading edge convention adopted by the American Society of Echocardiography. Digital images were analysed by using a HDI Lab (ATL, Philips, Bothell, WA, USA). Measurements were performed using Scion Image for Windows (Scion Corp). The investigator who conducted the echocardiography was blinded for the treatment status.

### Left ventricular pressure volume measurements

Invasive assessment of hemodynamics was performed under anaesthesia with isoflurane inhalation (initially 5%, after intubation 2% isoflurane). Mice were placed on controlled heating pads and mechanically ventilated. The right internal carotid artery was exposed, and a microtip catheter transducer (SPR-839, Millar Instruments, Houston, Tex, USA) was inserted into the right carotid artery and advanced into the left ventricle (LV) under pressure control. After stabilization, the pressure signals were recorded continuously with a pressure-volume conductance system (MPVS-300, Millar Instruments, Houston, Texas, USA) coupled with a PowerLab converter (PowerLab 4/20, ADInstruments Pty Ltd., Colorado Springs, CO, USA), stored by using suitable software (LabChart, ADInstruments), and displayed on a personal computer. PVAN software (Millar Instruments) was used for subsequent analysis of pressure-volume loops. The raw conductance volumes were corrected for parallel conductance by the hypertonic saline dilution method. For absolute volume measurements, the catheter was calibrated with known volumes of heparin-treated rat blood.

### Histopathology

After in vivo measurements, the heart was arrested in end-diastole by injection of 1ml of 1 mol/l KCl (B. Braun, Melsungen, Germany). Organs such as heart, lung and liver were excised, weighed, and frozen in liquid nitrogen. The heart was divided into the atria, left ventricle including the intraventricular septum, and the right ventricle. Histological studies were conducted using formalin-fixed, paraffin embedded hearts from animals of all groups. Cross sections of the LV obtained midway between base and apex were stained with haematoxylin/eosin and myocyte size was measured as cross sectional area using the ImageJ software (ImageJ, NIH, USA). Picrosirius red staining was performed to detect collagen deposition. Evidence of fibrosis was evaluated using the ImageJ software (ImageJ, NIH, USA) in light microscopy pictures. Collagen area fraction in percent was then calculated as collagen area to tissue area ratio. TUNEL staining (In Situ Cell Death Detection Kit, Roche) was used to quantify cardiomyocyte apoptosis rate in LV tissue slides. In cultured cardiomyocytes trypan blue staining (Sigma-Aldrich, Cat.-Nr. T8154) was performed to detect necrosis and apoptosis rates. For measuring capillary distribution in LV tissue we used haematoxylin/eosin staining and related the number of capillaries to tissue area.

Immunoflourescence was used to assess cytoskeleton structure and to quantify cell size in cultured cardiomyocytes. Phalloidin was used to stain actin filaments. Nuclei were counterstained with Hoechst 33342.

### Quantitative real-time PCR

Total RNA was isolated from mouse left ventricular tissue using the Trizol reagent (Invitrogen). cDNA was synthesized with Revert Aid First strand cDNA synthesis kit (Fermentas). Real-time polymerase chain reaction (PCR) was performed using the LightCycler® system (Roche Diagnostics, Mannheim, Germany) according to the manufacturer's instructions. All real-time PCR sample reactions were performed in triplicate and normalized to HPRT mRNA expression. Primers and specific probes were designed using the Universal Probe Library from Roche Diagnostics. The following primers were used: for HPRT 5′-GTCAAGGGGGACATAAAAG-3′ and 5′-TGCATTGTTTTACCAGTGTCAA-3′, probe # 22; for ANF 5′-CAACACAGATCTGATGGATTTCA-3′ and 5′-CCTCATCTTCTACCGGCATC-3′, probe # 25; for BNP 5′-GTCAGTCGCTTGGGCTGT-3′ and 5′-AGAGCTGGGGAAAGAAGAGC-3′, probe # 13. A standard curve was run with the dilution series of the amplified fragment allowing for mRNA copy number calculation.

### Immunoblot analyses

Immunoblot analyses were done for protein lysates prepared from the LV of wild type and transgenic mice (3 months unless otherwise stated) as well as for protein lysates prepared from primary cultures of ventricular cardiac myocytes. Protein concentration was measured using BCA protein assay (Interchim). Equal amounts of protein were separated with SDS-PAGE and transferred to a nitrocellulose membrane (Millipore). The membranes were incubated overnight at 4°C with primary antibody. Antibodies used were anti-eIF2Bε (Santa Cruz Biotechnology, Santa Cruz, CA, USA),. anti-GSK-3β (BD, Franklin Lakes, NJ, USA), anti-DYRK1A (Cell Signaling Technology, Beverly, MA, USA), anti-DYRK2 (Abcam, Cambridge, MA, USA), anti-eIF2α (Cell Signaling Technology), anti-Ubiquitin (Cell Signaling Technology), anti-α-Actinin (Sigma-Aldrich, St. Louis, MO, USA).Anti-rabbit IgG and anti-mouse IgG horseradish peroxidase-conjugated antibodies (Cell Signaling Technology) were used as secondary antibodies. Bands were quantified by densitometry using the Image J program.

### Co-Immunoprecipitation

Co-immunoprecipitation (Co-IP) was used to study protein-protein interactions by immunoprecipitating a specific target protein and determining what other proteins are co-precipitated. Protein lysates were prepared in Pierce IP lyses buffer (Thermo scientific). Tissue lysates were pre-cleared by adding G-Sepharose (Roche) followed by incubation with eIF2Bε monoclonal antibody (Santa Cruz) for 4 hours at 4°C. Immune complexes were pulled down by incubating with Protein G-Sepharose for 4 hours at 4°C followed by washing with ice-cold lyses buffer 4–5 times, to eliminate non-specific interactions. Protein G-Sepharose bound immune-complexes were then resuspended in Laemmli sample buffer, boiled for 5 min and analyzed by western blotting. In negative control, no primary antibody was added.

### Chronic β adrenergic stimulation via Isoproterenol

Mice were anesthetized with isoflurane, and Alzet osmotic minipumps (model 2002, Durect Corp, Cupertino, CA) were implanted subcutaneously in the neck during a brief surgery. Mice were randomized to receive isoproterenol HCl (I6504, Sigma-Aldrich, St. Louis, MO) at a dose of 30 µg/g per day dissolved in acidified isotonic saline, or isovolumic acidified saline alone (vehicle). Isoproterenol treatment was kept for 48 h respectively 14 days, as indicated.

### Mouse model of ascending aortic banding

Male C57BL/6 at the age of 10 weeks were used in this experiment. Mice were sedated preliminary with medetomidine 500 µg/kg i.p and then anesthetized with isoflurane inhalation (induction 3%, maintenance 2%) and buprenorphine 0.05-0.1 µg/g i.p. before endotracheal intubation. Mice were placed supine, and temperature was maintained at 37°C with a heating pad. A horizontal skin incision was made at the level of the suprasternal notch and the thyroid was retracted to allow visualization of the great vessels and upper part of the left atrial appendage. With a curved forceps the ascending portion of the aorta was dissected from the pulmonary trunk and a wire with a snare on the end was passed under the ascending aorta. A 6-0 silk suture was snared with the wire and pulled back around the aorta. A bent 25-gauge needle was then placed next to the aortic arch, and the suture was snugly tied around the needle and aorta. After ligation, the needle was quickly removed. The skin was closed, atipamezole 750 µg/kg was applied s.c. and mice were allowed to recover on a warming pad until they were fully awake.

### Primary culture of ventricular cardiac myocytes

Primary cultures of ventricular cardiac myocytes were prepared from 1–3-day old BR-Wistar rats (Charles River Laboratories) and purified using a discontinuous Percoll gradient. Cells were cultured in cardiac myocyte culture medium containing Dulbecco's modified Eagle's medium (DMEM)/F-12 supplemented with 5% horse serum, 4 µg/mL transferrin, 0.7 ng/mL sodium selenite, 2 g/L bovine serum albumin (fraction V), 3 mM pyruvic acid, 15 mM HEPES, 100 µM ascorbic acid, 100 µ/mL ampicillin, 5 µg/mL linoleic acid and 100 µM 5-bromo-20-deoxyuridine. We obtained cultures in which more than 95% of cells were myocytes. Culture medium was changed to serum-free at 24 h. The investigation was carried out in accordance with the Guide for the Care and Use of Laboratory Animals published by the US National Institutes of Health (NIH Publication No. 85-23, revised 1996) and was approved by the local animal ethics review board (Approval No. 40631).

### siRNA transfection

For at least 5 h myocytes were cultured in serum and antibiotic free medium. For transfection of 2×10^6^ cardiac myocytes in a 6 cm dish 250 pmol siRNA was diluted in 650 µL serum- and antibiotic free OptiMEM (Invitrogen). In another tube 4.5 µL of Lipofectamin 2000 (Invitrogen) was diluted in 200 µL OptiMEM and incubated for 5 min. After complex formation the solution and OptiMEM were added to the cells. The cells were incubated for 72 h at 37°C. In all siRNA mediated knockdown experiments myocytes transfected with non-specific (scrambled) siRNA were used as controls. siRNAs used were DYRK2 5-AGA-AUA-AUC-CAC-UGU-GAC-CUU-AAG-CCC-3 and non-specific control siRNA 5-AGG-UAG-UGU-AAU-CGC-CUU-GTT-3. siRNAs were synthesized by MWG (Ebersberg, Germany).

### Adenoviral infection

DYRK2 adenovirus (ABM Goods, Cat. Nr. 052224A) was used as vector to express DYRK2 protein in cardiomyocytes cultured in serum- and antibiotic free medium. Amplification was previously performed following manufacturer's protocol. After incubation for 72 h at 37°C cells were analysed using immunofluorescence or immunoblot analyses.

### Statistical analysis

Data are reported as mean ± SEM. Differences between groups were tested for statistical significance using unpaired 2 tailed- Student t-test or multiple analyses of variance, when applicable. SPSS V15.0 was used for statistical analysis. A *P-*value of <0.05 was considered significant. Error bars in figures indicate standard error if not indicated otherwise.

## Results

### Overexpression of eIF2Bε does not alter cardiac phenotype in vivo, but lack of phosphorylation by GSK-3β elicits left ventricular hypertrophy

**Figure 1:**
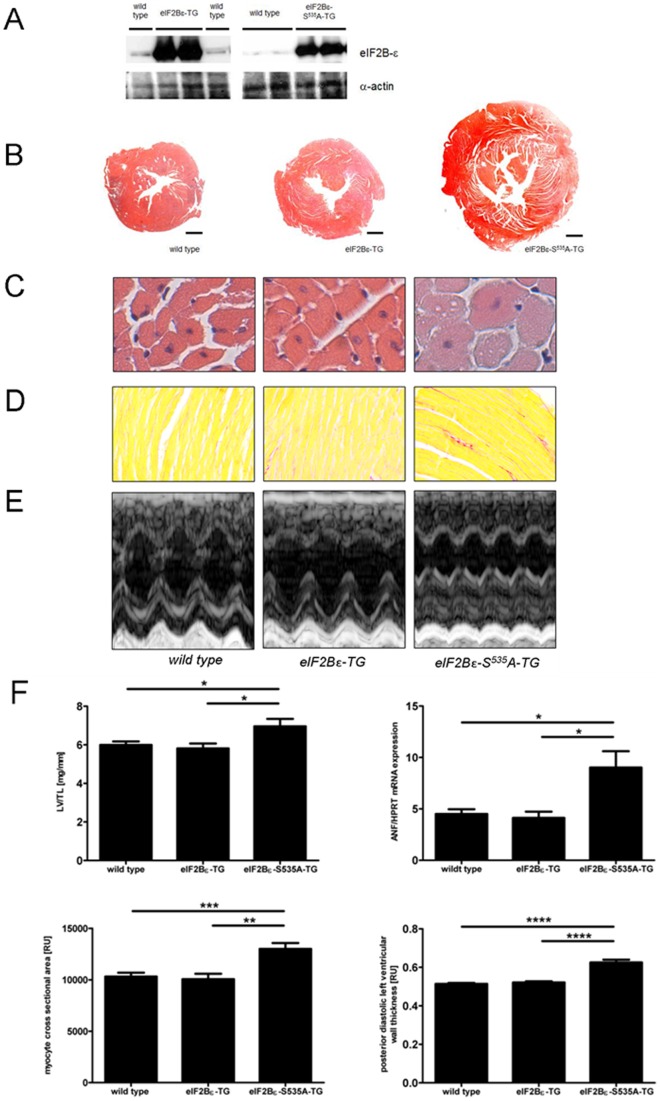
Baseline characterisation at the age of 3 months: eIF2Bε-S^535^A-TG develops spontaneous left ventricular hypertrophy. **A**: Overexpression of eiF2Bε at protein level in immunoblot analysis of left ventricular tissue. **B:** Exemplary transversal sections of left ventricles from wild type and transgenic animals. Bar 1 mm. **C:** Representative microphotographs of haematoxylin and eosin-stained myocardial tissue sections. **D:** Collagen deposition in LV tissue sections stained with Picrosirius Red. **E:** Examples of echocardiography of animal model studies. **F:** Collected data consistently shows hypertrophic phenotype in eIF2Bε-S^535^A-TG with increased LV weight to tibia length (n = 23 WT, n = 13 eIF2Bε-TG and n = 10 eIF2Bε-S^535^A-TG. * <0.05), elevated expression of hypertrophy markers (n = 13 WT, n = 6 eIF2Bε-TG and n = 8 eIF2Bε-S^535^A-TG * <0.05), increased myocyte cross sectional area (n = 20 WT, n = 15 eIF2Bε-TG and n = 9 eIF2Bε-S^535^A-TG. ** <0.01) and posterior LV wall thickness (n = 7 WT, n = 7 eIF2Bε-TG and n = 9 eIF2Bε-S^535^A-TG. *** <0.0001).

Confirmation of the overexpressions at protein level is shown in **Figure 1A**. Compared with C57BL/6 mice, eIF2Bε-TG and eIF2Bε-S^535^A-TG had normal litter sizes and showed no increased morbidity or mortality. Cardiac phenotyping was initiated at the age of 3–4 months. By contrast to our previous findings in vitro in cultured cardiac myocytes, overexpression of eIF2Bεin vivo did not lead to myocardial hypertrophy. In fact, eIF2Bε-TG animals displayed a cardiac phenotype comparable to wild type animals. However, baseline characterisation of eIF2Bε-S^535^A-TG revealed a spontaneous myocardial hypertrophy. Morphological characteristics for left ventricular hypertrophy in eIF2Bε-S^535^A-TG mice were already apparent to the eye in echocardiographic examination (**Figure 1E**) or when examining gross pathology of the excised hearts (**Figure 1B**) and could be verified consistently in all analyses (**Figure 1F**). Biometric as well as functional measurements are found in **Table** **1**. Systolic and diastolic function differed not significantly in both eIF2Bε-TG and eIF2Bε-S^535^A-TG compared to wild type. Histopathologic analysis revealed increased cardiomyocyte size in eIF2Bε-S^535^A-TG (**Figure 1C**; WT 10843±347 RU vs. eIF2Bε-TG 10940±829 RU vs. eIF2Bε-S^535^A-TG 13004±570 RU, p<0.01) accompanied with slightly increased collagen deposition in the left ventricle (**Figure 1D**, WT5.9±0.3% vs. eIF2Bε-TG 6.0±0.3% vs. eIF2Bε-S^535^A-TG 7.8±0.9%, p<0.01). In addition, no differences in apoptosis rate and capillarity have been observed between the groups. ANF and BNP as molecular markers of cardiac hypertrophy were clearly increased on mRNA level in eIF2Bε-S^535^A-TG (**Figure 1F**). Protein analysis of ubiquitinated proteins in left ventricular tissue showed a strong increase in eIF2Bε-S^535^A-TG, demonstrating elevated protein turnover (**Figure S1**). eIF2Bε-WT TG however, revealed an extent of Ubiquitin expression comparable to wild type.

**Table 1:** Biometric and functional measurements at the age of 3–4 months.

	wild type	eIF2Bε-TG	eIF2Bε-S^535^A-TG
Morphological parameters	n = 23	n = 13	n = 10
Heart/tibia length ratio [mg/mm]	8.0±0.2	7.9±0.3	10.0±0.5 (^a^p<0.001)
LV/tibia length ratio [mg/mm]	6.0±0.2	6.1±0.2	7.5±0.4 (^a^p<0.001)
RV/tibia length ratio [mg/mm]	1.3±0.1	1.2±0.1	1.8±0.1 (^a^p<0.001)
Lung/tibia length ratio [mg/mm]	8.2±0.4	8.1±0.4	7.4±0.2 (^a^p = n.s.)
Liver/tibia length ratio [mg/mm]	81.2±2.2	74.0±2.8	84.2±5.0 (^a^p = n.s.)

Echocardiographic parameters	n = 16	n = 7	n = 10
Heart rate [min^−1^]	459±12	470±30	500±9 (^a^p<0.05)
AWTD [mm]	0.48±0.01	0.51±0.01	0.65±0.02 (^a^p<0.001)
End-diastolic diameter [mm]	4.7±0.1	4.8±0.1	4.6±0.1 (^a^p = n.s.)
PWTD [mm]	0.50±0.01	0.52±0.01	0.66±0.02 (^a^p<0.001)
Ejection fraction [%]	76±1	76±1	74±1 (^a^p = n.s.)
Fractional shortening [%]	38±1	38±1	36±1 (^a^p = n.s.)

Hemodynamic parameters	n = 20	n = 12	n = 8
LVESP [mmHg]	83±2	89±2	84±4 (^a^p = n.s.)
LVEDP [mmHg]	4±0.5	5±0.5	5±0.3 (^a^p = n.s.)
Ejection fraction [%]	60±2	61±3	59±3 (^a^p = n.s.)
Stroke volume [µl]	21±1	22±2	25±2 (^a^p = n.s.)
LV dP/dt_max_ [mmHg/s]	9198±521	8604±738	9786±606 (^a^p = n.s.)
LV dP/dt_min_ [mmHg/s]	-8722±430	-8575±622	-8884±442 (^a^p = n.s.)
Tau _Weiss_ [ms]	5.1±0.3	5.8±1.7	4.7±0.2 (^a^p = n.s.)

AWTD: Diastolic anterior wall thickness; PWTD: Diastolic posterior wall thickness; LVESP: left ventricular end systolic pressure; LVEDP: left ventricular end diastolic pressure. All values of eIF2Bε-TG showed no significant difference to wild type. ^a^p  =  eIF2Bε-S^535^A-TG vs. wild type.

At the age of 20 months, wild type and eIF2Bε-TG showed, in comparable extent, a slight increase in diastolic anterior and posterior wall thickness and a moderate decrease of systolic function assessed by transthoracic echocardiography (EF and fractional shortening) in comparison to 4 months old animals. Aged eIF2Bε-S^535^A-TG, however, showed an accentuated increase in wall thickness and a pronounced loss in systolic function (**Figure S2**). However, there were no signs of cardiac decompensation in old eIF2Bε-S^535^A-TG, indicating still compensated myocardial hypertrophy. Expression of ANF on mRNA level as hypertrophy marker was still increased at the age of 20 months in eIF2Bε-S^535^A-TG in comparison to wild type or eIF2Bε-TG. Detailed biometric and functional data are outlined in **Table** **2**.

**Table 2:** Morphological and echocardiographic parameters at the age of 20 months.

	wild type	eIF2Bε-TG	eIF2Bε-S^535^A-TG
Morphological parameters	n = 9	n = 5	n = 9
Heart/tibia length ratio [mg/mm]	10.5±0.4	9.2±0.3	12.0±0.4 (^a^p<0.01)
LV/tibia length ratio [mg/mm]	7.8±0.4	6.6±0.3	8.8±0.3 (^a^p<0.05)
RV/tibia length ratio [mg/mm]	1.4±0.0	1.6±0.1	1.5±0.1 (^a^p = n.s.)
Lung/tibia length ratio [mg/mm]	11.0±0.4	11.6±0.4	13.1±2.1 (^a^p = n.s.)
Liver/tibia length ratio [mg/mm]	116.2±6.2	115.0±5.7	103.6±7.5 (^a^p = n.s.)

Echocardiographic parameters	n = 11	n = 5	n = 8
Heart rate [min^−1^]	481±11	487±18	473±10 (^a^p = n.s.)
AWTD [mm]	0.57±0.02	0.55±0.02	0.75±0.02 (^a^p<0.001)
End-diastolic diameter [mm]	5.2±0.1	4.7±0.1	5.1±0.2 (^a^p = n.s.)
PWTD [mm]	0.59±0.02	0.57±0.01	0.82±0.03 (^a^p<0.001)
Ejection fraction [%]	68±2	72±2	60±2 (^a^p<0.01)
Fractional shortening [%]	32±1	35±1	27±1 (^a^p<0.01)

All values of eIF2Bε-TG showed no significant difference to wild type. ^a^p  =  eIF2Bε-S^535^A-TG vs. wild type. AWTD: Diastolic anterior wall thickness; PWTD: Diastolic posterior wall thickness.

### Cardiac overexpression of eIF2Bε does not change the response of the heart to pathologic stress induced by chronic isoproterenol treatment

**Figure 2:**
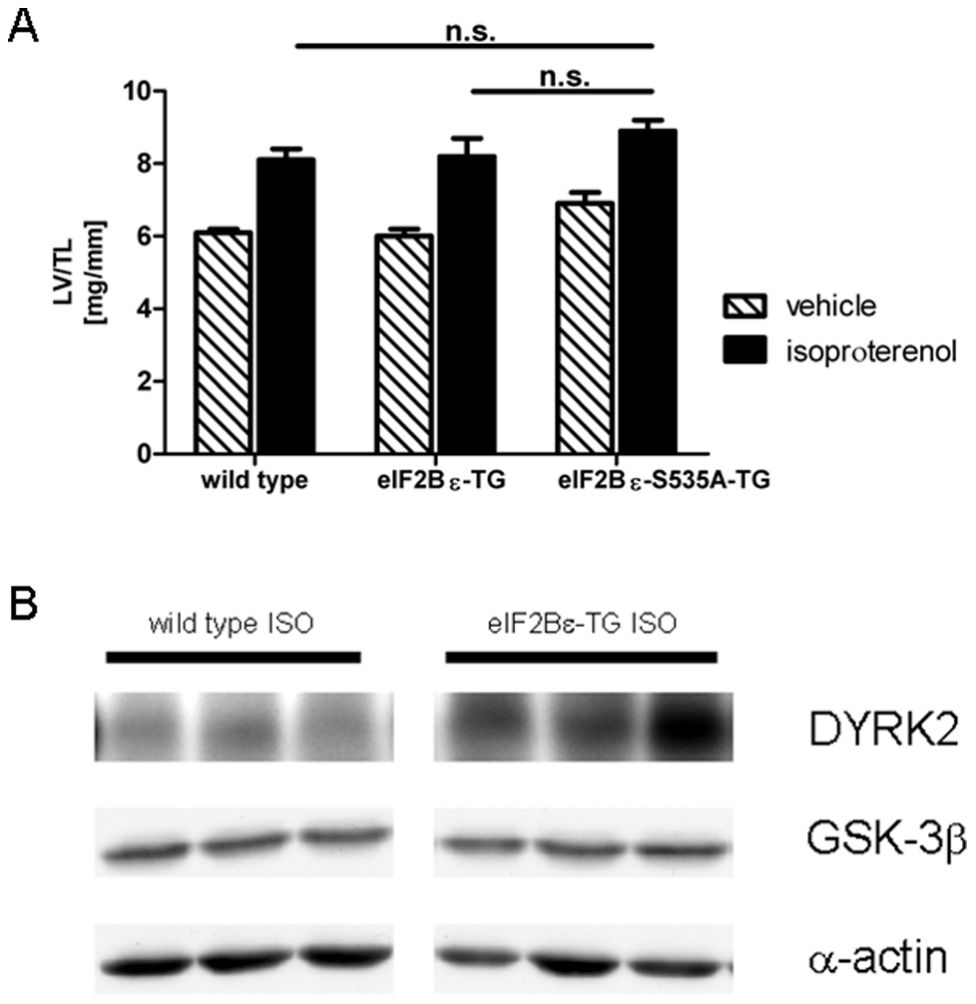
Cardiac overexpression of eIF2Bε does not change the response of the heart to pathologic stress induced by chronic isoproterenol treatment (30 µg/g per day for 14 days) (A). WT n = 13/14 for vehicle/ISO, eIF2Bε-TG n = 9/8 for vehicle/ISO and eIF2Bε-S^535^A-TG n = 8/8 for vehicle/ISO. **B:** Expression of DYRK2 is increased after chronic administration of isoproterenol in left ventricular tissue of eIF2Bε-TG. Sections are exemplary details of the same blot.

After 14 days of isoproterenol stimulation wild type mice developed myocardial hypertrophy associated with an increase of LV to tibia length ratio about 36% (8.1±0.3 mg/mm vs. 6.1±0.1 mg/mm, p<0.01) and an increase of cardiac myocyte size about 44% (13997±562 RU vs. 9699±585 RU, p<0.01) as compared to vehicle treated animals. Myocardial growth in eIF2Bε-TG occurred in a comparable extent (for LV/TL by 37%: 8.2±0.5 mg/mm vs. 6.0±0.2 mg/mm, p<0.01; for myocyte size by 41%: 13870±612 RU vs. 9847±912 RU, p<0.01; **Figure 2A**), indicating presence of active counterregulation mechanisms for increased protein levels of eIF2Bε even under hypertrophic stimulation. In eIF2Bε-S^535^A-TG, increase in LV to tibia length ratio and myocyte size was less distinct (for LV/TL by 28%: 8.9±0.3 mg/mm vs. 6.9±0.3 mg/mm, p<0.01; for myocyte size by 14%: 15534±589 RU vs. 13624±598 RU, p = n.s.) resulting in by trend still elevated values as compared to wild type or eIF2Bε-TG. However, LV weight to tibia length did not differ significantly anymore between the groups after chronic β adrenergic stimulation. For all biometric and functional data see **Table** **3**.

**Table 3:** Morphological and echocardiographic parameters after administration of isoproterenol or isovolumic vehicle for 14 days.

	wild type		eIF2Bε-TG		eIF2Bε-S^535^A-TG	
	isoproterenol	vehicle	isoproterenol	vehicle	isoproterenol	vehicle
Morphological parameters	n = 13	n = 14	n = 9	n = 8	n = 8	n = 8
Heart/tibia length ratio [mg/mm]	11.3±0.4	8.2±0.1 (^d^p<0.001)	11.5±0.6 (ap = n.s.)	8.0±0.3 (^e^p = 0.001)	11.9±0.3 (^b^p = n.s.; ^c^p = n.s.)	9.5±0.6 (^f^p<0.001)
LV/tibia length ratio [mg/mm]	8.1±0.3	6.1±0.1 (^d^p<0.001)	8.2±0.5 (ap = n.s.)	6.0±0.2 (^e^p<0.01)	8.9±0.3 (^b^p = n.s.; ^c^p = n.s.)	6.9±0.3 (^f^p<0.01)
RV/tibia length ratio [mg/mm]	2.1±0.2	1.4±0.1 (^d^p<0.01)	2.3±0.3 (ap = n.s.)	1.6±0.1 (^e^p<0.05)	1.8±0.1 (^b^p = n.s.; ^c^p = n.s.)	1.4±0.1 (^f^p<0.05)
Lung/tibia length ratio [mg/mm]	10.0±0.3	9.3±0.3 (^d^p = n.s.)	9.4±0.2 (ap = n.s.)	9.3±0.5 (^e^p = n.s.)	11.8±1.1 (^b^p<0.05; ^c^p<0.01)	8.5±0.5 (^f^p<0.01)
Liver/tibia length ratio [mg/mm]	101.2±3.9	95.6±3.5 (^d^p = n.s.)	102.9±3.9 (ap = n.s.)	85.9±3.3 (^e^p<0.05)	97.4±3.6 (^b^p = n.s.; ^c^p = n.s.)	104.9±3.3 (^f^p = n.s.)

Echocardiographic parameters	n = 11	n = 11	n = 9	n = 8	n = 7	n = 5
Heart rate [min^−1^]	556±10	478±21 (^d^p<0.01)	553±17 (ap = n.s.)	475±18 (^e^p<0.01)	508±23 (^b^p<0.05; ^c^p = n.s.)	507±25 (^f^p = n.s.)
AWTD [mm]	0.53±0.01	0.48±0.03 (^d^p = n.s.)	0.56±0.02 (ap = n.s.)	0.47±0.02 (^e^p<0.05)	0.57±0.02 (^b^p = n.s.; ^c^p = n.s.)	0.56±0.02 (^f^p = n.s.)
End-diastolic diameter [mm]	5.3±0.2	4.9±0.1 (^d^p<0.05)	5.1±0.1 (ap = n.s.)	4.5±0.1 (^e^p<0.05)	5.3±0.1 (^b^p = n.s.; ^c^p = n.s.)	4.7±0.2 (^f^p<0.05)
PWTD [mm]	0.57±0.01	0.50±0.02 (^d^p<0.01)	0.59±0.01 (ap = n.s.)	0.47±0.02 (^e^p = 0.001)	0.58±0.03 (^b^p = n.s.; ^c^p = n.s.)	0.57±0.03 (^f^p = n.s.)
Ejection fraction [%]	75±2	72±2 (^d^p = n.s.)	77±1 (ap = n.s.)	75±3 (^e^p = n.s.)	72±2 (^b^p = n.s.; ^c^p = n.s.)	71±4 (^f^p = n.s.)
Fractional shortening [%]	35±2	35±1 (^d^p = n.s.)	39±1 (ap<0.05)	38±3 (^e^p = n.s.)	35±1 (^b^p = n.s.; ^c^p = n.s.)	34±3 (^f^p = n.s.)

AWTD: Diastolic anterior wall thickness; PWTD: Diastolic posterior wall thickness.

a p  =  isoproterenol: wild type vs eIF2Bε-TG; ^b^p  =  isoproterenol: wild type vs eIF2Bε-S^535^A-TG; ^c^p  =  isoproterenol: eIF2Bε-TG vs eIF2Bε-S^535^A-TG. ^d^p  =  wild type: isoproterenol vs. vehicle; ^e^p  =  eIF2Bε-TG: isoproterenol vs. vehicle; ^f^p  =  eIF2Bε-S^535^A-TG: isoproterenol vs. vehicle.

### DYRK2 levels correlate strictly with phosphorylation of eIF2Bε by GSK-3β

**Figure 3:**
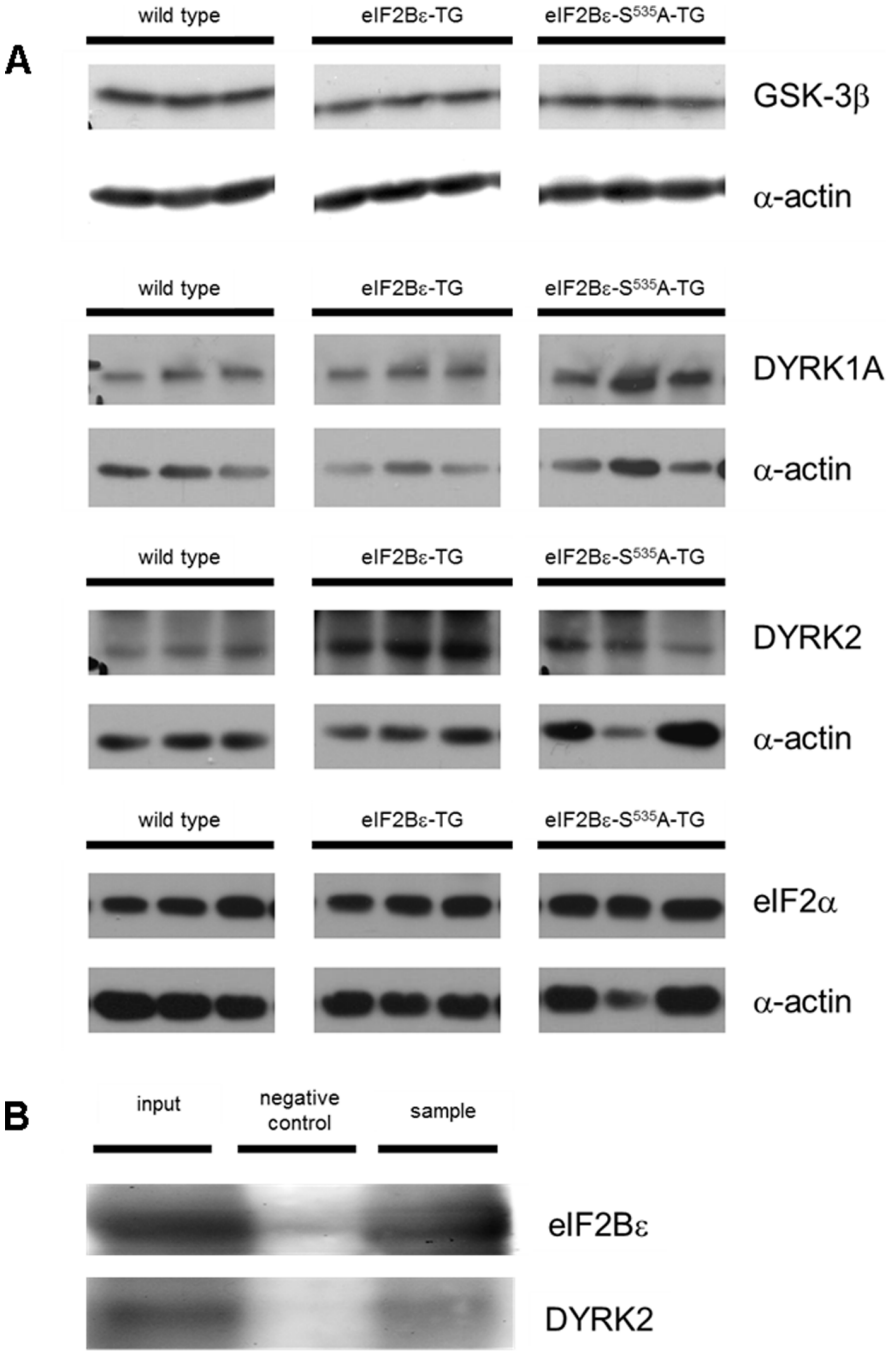
Expression of DYRK2 is elevated in left ventricular tissue in eIF2Bε-TG. GSK-3β, DYRK1A and eIF2α were not different between groups. In each case, sections are exemplary details of the same blot **(A)**. **B:** Immunoblots of co-immunoprecipitation demonstrating physical interaction of eIF2Bε and DYRK2 in left ventricular tissue of eIF2Bε-TG and eIF2Bε-S^535^A-TG.

In conclusion of our results, we expected GSK-3βto be the key regulator controlling the activity of eIF2Bε overexpression, since a loss of its phosphorylation site in eIF2Bε-S^535^A-TG animals lead to spontaneous cardiac hypertrophy. However, protein analysis of GSK-3β in left ventricular tissue did not show any differential expression or changes in phosphorylation status between the groups (**Figure 3A**). Faced with this surprising result, we were searching for alternative regulating mechanisms.

Four protein kinases have been previously described in vitro to directly phosphorylate eIF2Bε and thereby have potential regulating action: Caseinkinase 1 and 2 (CK1 and CK2), Dual-specificity tyrosine phosphorylated and regulated kinase 1A and 2 (DYRK1A and DYRK2) and Glycogen synthase kinase 3β (GSK-3β) [10], [11].

We observed in this study, that increased expression of eIF2Bε in eIF2Bε-TG animals was accompanied by increased levels of DYRK2under baseline conditions (**Figure 3A**) as well as under chronic administration of isoproterenol (**Figure 2B**).All other known phosphorylating kinases of eIF2Bεdidn't show any differential expression between the groups (**Figure S2B**). As mentioned before it was previously shown in vitro that the activity of GSK-3β is known to be mediated by DYRK2, suggesting a priming mechanism that makes eIF2Bε more susceptible for phosphorylation by GSK-3β. To evaluate a possible impact of other pathways in controlling eIF2Bε overexpression we analyzed expression and phosphorylation levels of kinases known to regulate protein synthesis. The activator of protein translation mTOR and its downstream targets 4E-BP and P70S6 were not differentially expressed in transgenic mice (Supplementary Figure 4).

**Figure 4:**
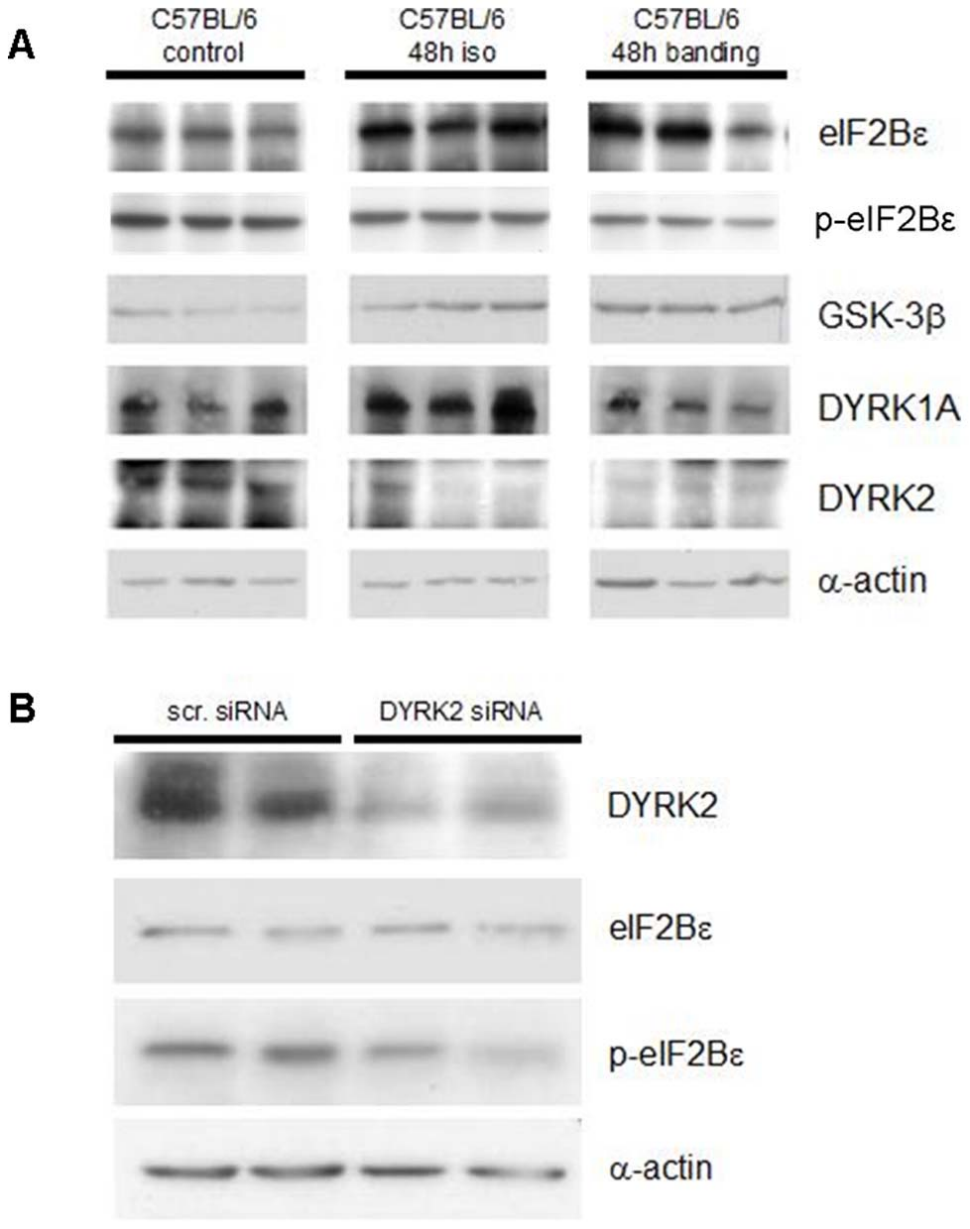
Levels of phospho(S535)-eIF2Bε and DYRK2 expression show strict correlation, indicating a coordinated response pattern following pathologic injury. **A:** In acute myocardial response 48 h after hypertrophic stimulation by isoproterenol or aortic banding of C57BL/6 wild type mice eIF2Bε levels were elevated whereas phospho(S535)-eIF2Bε and DYRK2 levels were significantly decreased. **B:** siRNA mediated knockdown of DYRK2 lead to reduced phospho(S535)-eIF2Bε levels in vitro. Exemplary immunoblots of three individual experiments.

Consistently throughout our experiments we could prove a strict positive correlation of DYRK2 levels and phosphorylation of eIF2Bε by GSK3-β on serine 535.

Following hypertrophic stimulation of C57BL/6 wildtype mice, eIF2Bε was higher expressed after 48 h but less phosphorylated by GSK-3β, which was consistently accompanied by a clearly decreased DYRK2 expression (**Figure 4A**). After 4 weeks, in a chronic compensated hypertrophic state the expression of eIF2Bε and DYRK2 returned to baseline levels both after isoproterenol treatment and aortic banding (data not shown).

Analysis of protein expression of eIF2Bε under conditions of heart failure in a swine model of atrial fibrillation (AF) revealed an unchanged expression of eIF2Bε. However, p-eIF2Bε(S535) was reduced (**Figure S3**), supporting again a regulatory role for modulating activity of eIF2Bε in response to cardiac stresses.

**Figure 5:**
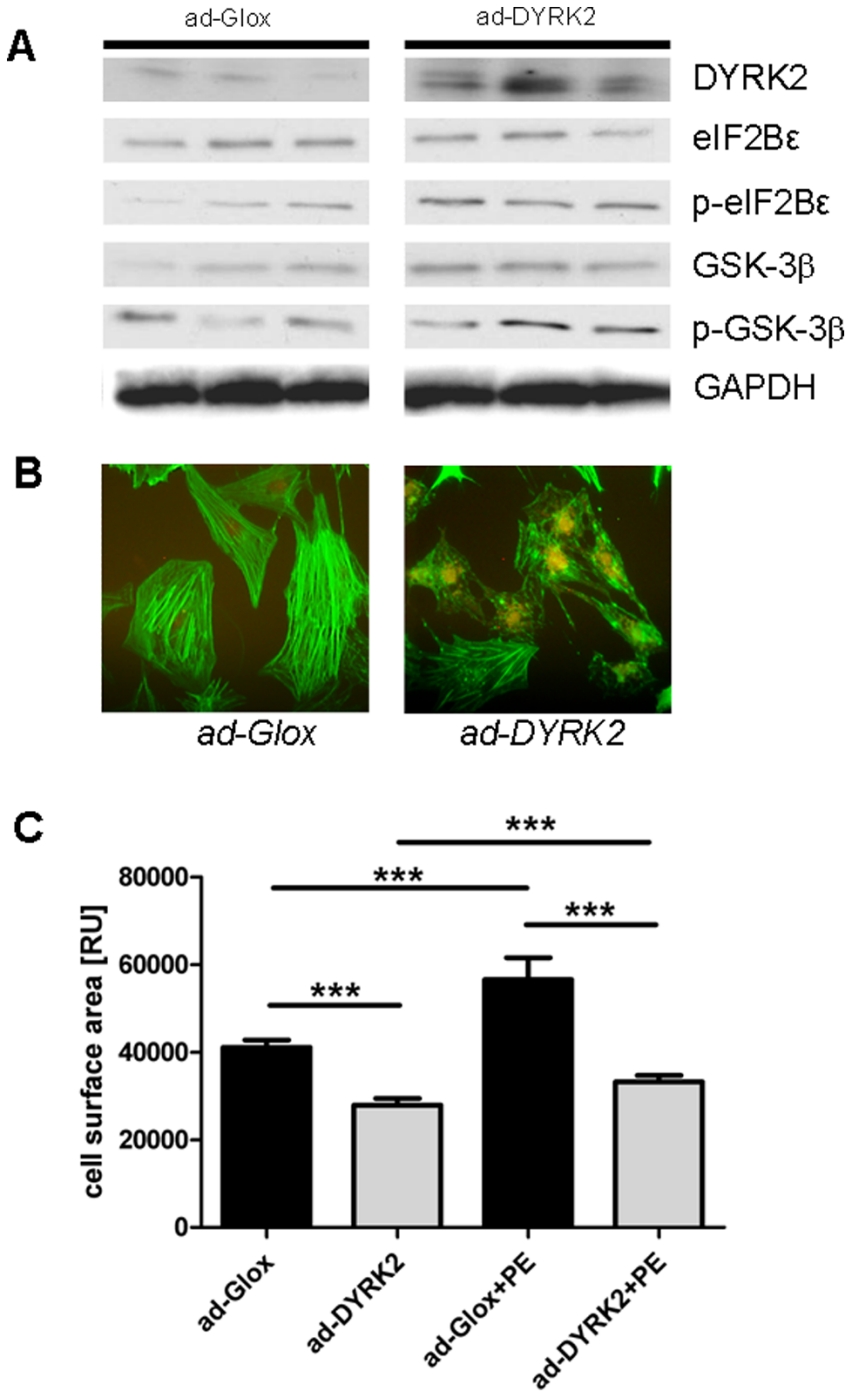
Adenoviral induced overexpression of DYRK2 in cultivated cardiomyocytes reduces baseline cell surface area and diminishes hypertrophic response to phenylephrine stimulation. **A:** Overexpression of DYRK2 is accompanied by increased levels of phospho(S535)-eIF2Bε, and GSK-3β. **B:** Representative cultivated cardiomyocytes with reduced cell size and density of cytoskeleton following DYRK2 overexpression under baseline conditions. **C:** Cardiomyocyte size was significantly reduced in DYRK2 overexpressing cells compared to negative control under baseline conditions. Further, DYRK2 overexpression could almost inhibit the hypertrophic response to phenylephrine stimulation (n = 117 ad-Glox, n = 125 ad-DYRK2, n = 51 ad-Glox+PE, n = 68 ad-DYRK2+PE, *** <0.0001).

Further, after siRNA mediated knockdown of DYRK2 phosphorylation of eIF2Bε was reduced (**Figure 4B**) whereas it was increased after adenoviral induced overexpression of DYRK2 in cultivated cardiomyocytes (**Figure 5A**).

Additionally, direct physical interaction of eIF2Bε and DYRK2 was proven by co-immunoprecipitation using left ventricular tissue lysates from eIF2Bε-TG and eIF2Bε-S^535^A-TG (**Figure 3B**). Protein expression of DYRK1A and eIF2α did not differ in all groups.

### DYRK2 overexpression leads to spontaneously reduced cardiomyocyte size and diminishes hypertrophic response

In order to prove our hypothesis, that DYRK2 acts as a negative regulator of cardiomyocyte hypertrophy by priming inhibitory activity of GSK-3β on eIF2Bε we induced adenoviral overexpression of DYRK2 in cultivated cardiomyocytes.

We observed a significantly reduced cardiomyocyte size following DYRK2 overexpression compared to Glox infected cardiomyocytes serving as negative control (ad-Glox 41,117±1641 vs. ad-DYRK2 27,877±1580, p<0.0001, **Figure 5B and 5C**).Further, DYRK2 overexpression could nearly inhibit hypertrophic response to β-adrenergic stimulation by phenylephrine (ad-Glox+PE 56,567±5000 vs. ad-DYRK2+PE 33,251±1470, p<0.0001, **Figure 5C**).

The analysis of signalling pathways revealed an increased phosphorylation of eIF2Bε on Serine 535 by GSK-3β under conditions of DYRK2 overexpression in vitro (**Figure 5A**).

## Discussion

Control of protein translation machinery is a fundamental regulatory mechanism in development of myocardial hypertrophic response. In this study we show that in vivo the activity of the ε-subunit of Eukaryotic initiation factor 2B (eIF2Bε) underlies in the heart a tight regulatory network by interaction of GSK-3β and its priming kinase DYRK2. However, the release of their inhibitory influence in eIF2Bε-S^535^A-TG mice induces myocardial hypertrophy by increasing protein synthesis. Thereby we identify the Dual specificity tyrosine-phosphorylation-regulated kinase 2 (DYRK2) to regulate the repressive activities of GSK-3β on eIF2Bε, which determine the response of the heart upon pathologic injury.

### Phosphorylation of eIF2Bε on Serine 535 by GSK-3β is required for controlling its prohypertrophic activity in vivo

By mediating the guanine nucleotide exchange, eIF2Bε regulates the initiation of mRNA translation in all eukaryotic cells. We have previously shown that phosphorylation of eIF2Bε on serine 535 by GSK-3β controls cardiomyocyte hypertrophy following β-adrenergic stimulation in cultured cardiomyocytes [3]. In line with these previous findings, disinhibition of eIF2Bε from the influence of GSK-3β in eIF2Bε-S^535^A-TG mutant animals showed a distinct hypertrophic phenotype, which aggravated with aging and was associated with a trend for exaggerated hypertrophic response upon pathological stimulation. However, it was very surprising to see that, in contrast to our previously reported findings in cultured cardiomyocytes, even a very robust cardiac overexpression of the wild type form of eIF2Bε did neither change left ventricular hypertrophy on the organ or cellular level nor any of the classical hypertrophic marker genes. Moreover and even more unexpectedly, cardiac overexpression of eIF2Bε did not change minutely the response of the heart to pathologic stress, suggesting that a so far unrecognized and potent counter regulatory mechanism, which was not obvious in our previously reported cell culture system [3], controls activity of eIF2Bε in vivo.

Our data shows that the activity of eIF2Bε in vivo is strictly dependent on the ability of GSK-3β to phosphorylate serine 535 (corresponds to serine 540 in the human sequence). If serine 535 cannot be phosphorylated, the activity of eIF2Bε cannot be controlled under overexpression conditions. Conversely, serine 535 phosphorylation can compensate even for a strong overexpression of eIF2Bε. It is interesting to note that overexpression of eIF2Bε led to spontaneous hypertrophy in cultured cardiac myocytes [3], while no spontaneous phenotype developed in vivo not even on the long term after 20 months of age, indicating that in this case counteracting mechanisms are more powerful on the whole organism level. Anyhow, it is of note that neither expression nor phosphorylation status of GSK-3β -the prime candidate molecule to control activity of eIF2Bε- was not altered in the eIF2Bε-TG animals suggesting that another signalling molecule is the crucial factor for this observation.

### DYRK2 acts as a negative regulator of cardiomyocyte growth by coordinating the repressor function of GSK-3β on eIF2Bε

In search for the missing link of our observation and given the limited number of in vitro known interaction partners of eIF2Bε, we recapitulated that there is evidence that GSK-3β is only able to phosphorylate eIF2Bε if serine 539 is already phosphorylated by another kinase (reviewed in [14]). In this context, the kinases DYRK1A and DYRK2 have been both described in vitro to phosphorylate eIF2Bε at serine 539 and to act as a priming kinase for GSK-3β [11]. While the extent of protein expression of DYRK1A, which has recently been identified to be a negative regulator of cardiac hypertrophy [15], was not changed in our transgenic model, we observed a concomitant upregulation of DYRK2 in animals overexpressing eIF2Bε. Furthermore, also under β-adrenergic stimulation with isoproterenol an upregulation of DYRK2 could be observed. We did not observe any changes in expression of CK1 and CK2 or eIF2 (αP) in our transgenic model, but strictly correlating levels of DYRK2 and phosphorylation of eIF2Bε by GSK-3β in acute hypertrophic reaction in wildtype mice. A specific regulatory circuit is further supported by the fact that siRNA mediated knockdown of DYRK2 resulted in a decreased expression of p-eIF2Bε(S535), as to expect in line with less activity of DYRK2 acting as priming kinase for GSK-3β. Corresponding results can be reported following adenoviral induced overexpression of DYRK2 which was accompanied by elevated levels of p-eIF2Bε(S535). Physical interaction of eIF2Bε and DYRK2 was proven by co-immunoprecipitation using left ventricular tissue lysates from our transgenic animals. In conclusion, these results support a coordinated response pattern with interaction of GSK-3β and its priming kinase DYRK2 phosphorylating eIF2Bε. In order to prove functional relevance of these regulatory axes we evaluated the effects on cardiomyocyte growth. Following adenoviral overexpression of DYRK2 we observed significantly reduced cardiomyocyte size under baseline conditions. Moreover DYRK2 overexpression had antihypertrophic effect and leads to reduced cardiomyocyte growth as response to phenylephrine stimulation. To complete our understanding of involved signalling pathways in regulating eIF2Bε overexpression, we analysed several main regulators of protein synthesis (Figure S4). The result that none of them was differentially expressed emphasizes for the first time the predominating role of DYRK2 as negative regulator of cardiomyocyte growth.

### DYRK2 may play a physiological role in mediating cardiomyocyte hypertrophy in vivo

We could demonstrate in C57BL/6 wild type mice that phosphorylation of eIF2Bε by GSK-3β and DYRK2 expression are upregulated under conditions of increased protein translational demand, such as acute hypertrophic response. In the state of chronic compensation both levels return to baseline. In conclusion with our in vitro findings, the correlation of DYRK2 and phospho-eIF2Bε with conditions of increased protein synthesis are indicative for a physiological role of this regulatory axis in cardiac hypertrophy. Further evaluation of this hypothesis is necessary but goes beyond the scope of this investigation.

### Protein translation as a potential target for treating LVH

Hypertrophic growth of cardiomyocytes is accompanied by increases in both protein synthesis and proteolysis rates; however the more rapid rate of protein synthesis relative to protein breakdown results in a net increase in protein accumulation and myocyte mass [16]. Increases in cellular protein synthesis rates can be due to a general augmentation of transcription, to an increase in post-transcriptional rates (i.e. splicing) or to accelerated translation rates [17]. Most proteins of the “fetal gene program” (including atrial natriuretic peptide (ANF), B-type natriuretic peptide, α-actin, myosin heavy chain (MHC) and others) are known to be regulated at the level of transcription [17]. Regarding the global increases in protein synthesis observed during cardiac hypertrophy, it is most likely that synthesis rates are regulated at the level of mRNA translation, as the availability of mRNA is not generally limited for increases in overall cell protein synthesis during growth [16]. The mRNA translation process can be boosted at the level of translation efficiency (i.e. the efficiency of translation of existing ribosomes) and of translation capacity (i.e. the amount of ribosomes actively translating mRNAs) [17]. During the first phase of hypertrophic growth, the limiting step for the rise is the increase of translation initiation [18] which emphasizes again the potential role of eIF2Bε/DYRK2-axisas an antihypertrophic target.

Deregulation of the translational machinery is an important fundament of numerous human diseases as diverse as tissue hypertrophy, cancer, neurodegeneration and inflammation. Anyhow, no therapeutic option has been derived from this insight so far. Unravelling the mechanisms and control of mRNA translation will ultimately open up new therapeutic approaches for LVH, potentially involving a temporally and spatially restricted modulation of the activities of eIF2Bε and DYRK2 in the myocardium.

## Supporting Information

Figure S1 **Protein expression of Ubiquitin is increased in left ventricular tissue of eIF2Bε-S^535^A-TG, whereas eIF2Bε-TG shows a comparable Ubiquitin expression to wild types.**
(TIF)

Figure S2 **eIF2Bε-S^535^A-TG showed a pronounced loss of EF in echocardiography at the age of 20 months compared to 4 months.** (n = 11 WT, n = 5 eIF2Bε-TG, n = 8 eIF2Bε-S^535^A-TG. * <0.05).
(TIF)

Figure S3 **p-eIF2Bε is reduced under condition of heart failure in a swine model of atrial fibrillation (AF).** AF was induced by pacemaker stimulation. The model and details about pacemaker implantation and further experimental settings have been described previously [19].
(TIF)

Figure S4 **In order to explore other potential regulative mechanisms of eIF2Bε overexpression we analysed pathways known to have impact on protein synthesis rate.** Immunoblot analyses are shown above. Neither expression nor phosphorylation levels of these kinases differed between the groups.
(TIF)
