# Supplementary figures and images for: DYRK2 Negatively Regulates Cardiomyocyte Growth by Mediating Repressor Function of GSK-3β on eIF2Bε
Source: PLoS One. 2013 Sep 4;8(9):e70848. doi: 10.1371/journal.pone.0070848 (PMC3762802; doi:10.1371/journal.pone.0070848)

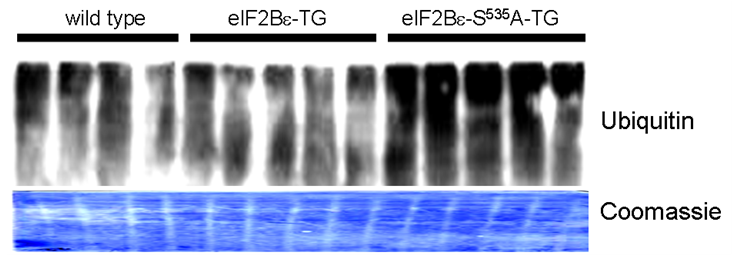

Supplement: Figure S1 — Protein expression of Ubiquitin is increased in left ventricular tissue of eIF2Bε-S535A-TG, whereas eIF2Bε-TG shows a comparable Ubiquitin expression to wild types. (TIF) [file pone.0070848.s001.tif]

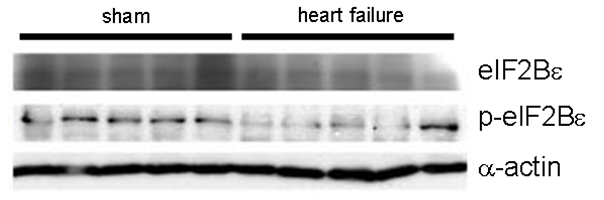

Supplement: Figure S3 — p-eIF2Bε is reduced under condition of heart failure in a swine model of atrial fibrillation (AF). AF was induced by pacemaker stimulation. The model and details about pacemaker implantation and further experimental settings have been described previously [19]. (TIF) [file pone.0070848.s003.tif]

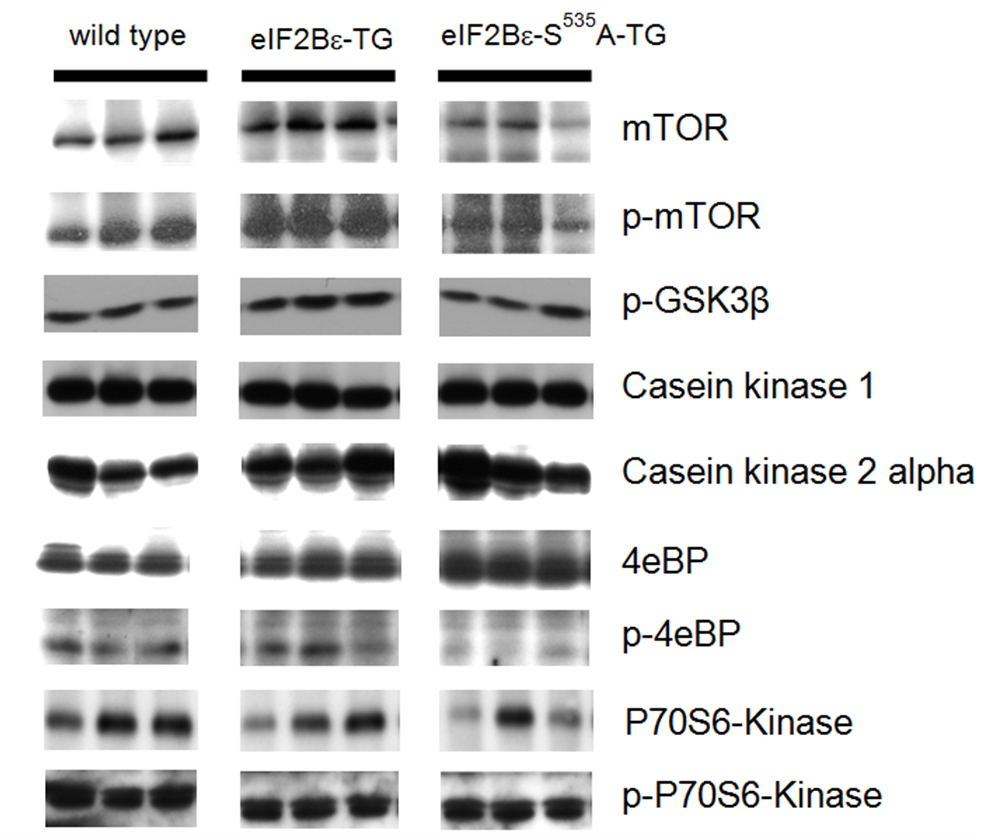

Supplement: Figure S4 — In order to explore other potential regulative mechanisms of eIF2Bε overexpression we analysed pathways known to have impact on protein synthesis rate. Immunoblot analyses are shown above. Neither expression nor phosphorylation levels of these kinases differed between the groups. (TIF) [file pone.0070848.s004.tif]
